# DNA methylation profiles delineate epigenetic heterogeneity in seminoma and non-seminoma

**DOI:** 10.1038/bjc.2011.468

**Published:** 2011-11-08

**Authors:** M Brait, L Maldonado, S Begum, M Loyo, D Wehle, F F Tavora, L H J Looijenga, J Kowalski, Z Zhang, E Rosenbaum, S Halachmi, G J Netto, M O Hoque

**Affiliations:** 1 Department of Otolaryngology–Head and Neck Surgery, Johns Hopkins School of Medicine, Baltimore, MD 21205, USA; 2 Department of Pathology, Johns Hopkins Medical Institutions, Baltimore, MD, USA; 3 Erasmus MC—University Medical Center Rotterdam, Josephine Nefkens Institute (Daniel den Hoed Cancer Center), Department of Pathology, Rotterdam, The Netherlands; 4 Division of Biostatistics/Department of Oncology, Johns Hopkins School of Medicine, Baltimore, MD, USA; 5 Biostatistics and Bioinformatics, Winship Cancer Institute of Emory University, Atlanta, GA, USA; 6 Davidoff Cancer Center, Rabin Medical Center, Petach Tikva, Israel; 7 Department of Urology, Bnai Zion Medical Center, Haifa, Israel

**Keywords:** testicular cancer, DNA methylation, epigenetics, biomarker, seminoma, non-seminoma

## Abstract

**Background:**

It remains important to understand the biology and identify biomarkers for less studied cancers like testicular cancer. The purpose of this study was to determine the methylation frequency of several cancer-related genes in different histological types of testicular cancer and normal testis tissues (NT).

**Methods:**

DNA was isolated from 43 seminomas (SEs), 14 non-SEs (NSEs) and 23 NT, and was assayed for promoter methylation status of 15 genes by quantitative methylation-specific PCR. The methylation status was evaluated for an association with cancer, and between SEs and NSEs.

**Results:**

We found differential methylation pattern in SEs and NSEs. *MGMT*, *VGF*, *ER-β* and *FKBP4* were predominately methylated in NSEs compared with SEs. *APC* and *hMLH1* are shown to be significantly more methylated in both subtypes in comparison with NT. When combining *APC*, *hMLH1*, *ER-β* and *FKBP4*, it is possible to identify 86% of the NSEs, whereas only 7% of the SEs.

**Conclusions:**

Our results indicate that the methylation profile of cancer-associated genes in testicular cancer correlates with histological types and show cancer-specific pattern for certain genes. Further methylation analysis, in a larger cohort is needed to elucidate their role in testicular cancer development and potential for therapy, early detection and disease monitoring.

Testicular cancer is the most commonly diagnosed malignancy among young men aged 15 to 40 years, and its incidence has doubled in the past 40 years (Chia *et al*, 2010). An annual increase of 3–6% is reported for Caucasian populations. In the United States, approximately 8500 newly diagnosed testicular cancer cases and 350 deaths were expected in 2010 (Jemal *et al*, 2010). Testicular germ cell tumours (TGCTs) represent over 95% of the testicular cancers and histopathologically are classified into two major groups of seminomas (SE) or non-SEs (NSE), frequently occurring as mixed tumours (Holmes *et al*, 2008). Histologically, SE resembles primordial germ cells/gonocytes, whereas NSE shows somatic, primitive embryonal or extra-embryonal differentiation (Horwich *et al*, 2006). Carcinoma *in situ* or intra-tubular germ cell neoplasia unclassified are believed to be the origin of both SE and NSE (Rajpert-De Meyts, 2006). They have a primordial germ cell/gonocyte origin, and it is important to emphasise that epigenetic reprogramming is known to occur during germ cells development.

The treatment of testicular cancer includes orchiectomy, and according to the metastatic condition surveillance, or adjuvant retroperitoneal surgery, radiation and chemotherapy are offered. The decision whether to choose the adjuvant treatment regimen is based only on clinical parameters, leading to around 25% development of metastasis in patients on surveillance or unnecessary adjuvant treatments in 20% of the patients. Genetic understanding of each tumour will enable to better tailor the treatment.

The importance of epigenetic alterations has long been demonstrated in carcinogenesis. Several studies have shown that methylation-associated silencing inactivates certain tumour suppressor genes (TSGs) as effectively as mutations and is one of the cancer-predisposing hits described in Knudson's two hit hypothesis. Promoter methylation, the most studied epigenetic alteration, is also increasingly recognised as a major mechanism of gene inactivation during TGCT progression (Manton *et al*, 2005; Ellinger *et al*, 2009). CG dinucleotide-rich regions, also known as CpG islands, in or near the proximal promoter regions of genes are targets for DNA methylation, leading to effective transcriptional silencing (Herman, 1999). In normal cells, CpG methylation is an important mechanism for regulating gene expression, whereas in cancer cells, aberrant promoter methylation (hypermethylation) can lead to abnormal gene silencing, including repression of TSGs. Another relevant aspect is that environmental and endogenous conditions can influence the epigenetic processes. The exposure to certain environmental risk factors may be related to the onset of cancer or to participate in carcinogenesis (Brait *et al*, 2009). There are many evidences that endogenous factors (such as oestrogens or androgens inhibitors) exposure may lead to cancer (Godmann *et al*, 2009), and endogenous factors (like hormonal stimuli) induce methylation of promoter region of certain genes (Kutanzi *et al*, 2010). Even though TGCT has high survival rates due to good responses to therapy, significant consequences of multimodality therapies exist with regard to general health, secondary late malignancies, reproduction and economic productivity (Sokoloff *et al*, 2007). A great need for understanding TGCT biology still exists to help curb its increasing incidence and potentially adopt effective prevention strategies.

Several genetic alterations have been shown in TGCTs. For example, abnormalities in the short arm of chromosome 12 (Mostert *et al*, 1998), as well as loss on chromosomes 1, 3, 5, 9, 11, 12q, 13q, 17p and 18q (Mathew *et al*, 1994; Lothe *et al*, 1995; al-Jehani *et al*, 1995; Honorio *et al*, 2003; Oosterhuis and Looijenga, 2005) have been reported. But major TSGs having a role in TGCTs are yet to be identified (Honorio *et al*, 2003). One of the most intriguing questions in the biology of TGCTs is how such distinct histological tumour subtypes (SE and NSE) can arise from the same cell type (cell of origin). Both subtypes exhibit similar cytogenetic abnormalities (Lutzker and Barnard, 1998). So far, molecular alterations that could distinct SEs from NSEs have not been clear yet. A pioneer study, performed in 1991, has shown that hypermethylation was abundant in NSEs, but not in SEs (Peltomaki, 1991). In 2002, Smiraglia *et al* (2002) demonstrated significant epigenetic differences between SEs and NSEs by a global methylation approach. In addition, studies of the X chromosome have demonstrated little or no methylation in SEs, and increased methylation in NSEs, particularly in more highly differentiated NSEs (Looijenga *et al*, 1997). Netto *et al* (2008) analysed global methylation status by immunohistochemistry and concluded that SEs cells generally retain the lack of methylation that occurs due to normal developmental erasure of methylation marks, whereas NSEs do not, thus showing more methylation. This was independently shown as well (Wermann *et al*, 2010).

In the present study, we used a candidate gene approach to investigate the methylation profile of 57 primary TGCT (of which 43 were SEs and 14 were NSEs) and 23 normal testis (NT) by quantitative methylation-specific PCR (QMSP). QMSP has been successfully used in other tumour models and has the benefit of providing accurate and precise data regarding the level of methylation in the various tumours. Six of the genes we evaluated, including *ARF*, *APC*, *MGMT*, *RAR-β2*, *CCNA1* and *hMLH1*, were previously shown to be aberrantly methylated in TGCT (Muller *et al*, 2000; Koul *et al*, 2002; Honorio *et al*, 2003; Olasz *et al*, 2005; Lind *et al*, 2007). The remaining nine genes we studied has been found to be methylated in other cancer types, but not yet tested in TGCT; these genes include *AIM1*, *PGP9.5*, *S100A2*, *ER-α*, *ER-β*, *MCAM*, *VGF*, *FKBP4* and *SSBP2.* Among the later genes, four genes (*MCAM*, *VGF*, *FKBP4* and *SSBP2*) were recently discovered by our group, using a pharmacological unmasking strategy in other cancer types (Hoque *et al*, 2008). We compared the promoter methylation profiles of SEs and NSEs along with NT to better understand the role of epigenetic silencing in testis tumourigenesis. Relationships between methylation values and clinicopathological parameters were further assessed.

## Materials and methods

### Study cohort

The TGCT and NT samples were retrospectively collected from the Johns Hopkins Medical Institutions tissue archive. To be included in the cohort, an eligible patient had to have a confirmed diagnosis of TGCT (or normal tissue) and a sufficient amount of archived tumour (or normal) material to allow for DNA extraction (tissue preserved in sectioned blocks; >50% of tumour cells). A total of 75% (43 out of 57) of our TGCT samples were SEs and 25% (14 out of 57) were NSEs. We also analysed 23 NT from archived samples (testis removed on castration procedures). Thus, a total of 80 samples (57 tumours and 23 normals) were tested for methylation pattern by QMSP. Demographic and clinical information was obtained from the computerised tumour registry at the Johns Hopkins Healthcare System. The tumours were classified according to the WHO Classification of Tumours, Pathology and Genetics of the Urinary System and Male Genital Organs (Looijenga, 2009). Patient characteristics included in this study are summarised in Table 1. Approval for research on human subjects was obtained from the Johns Hopkins University Institutional Review Boards. This study qualified for exemption under the US Department of Health and Human Services policy for protection of human subjects (45 CFR 46.101(b)) (IRB 03-11-12-06e).

### Gene selection

A total of 15 genes were selected for promoter methylation status analysis. Among these genes, six were previously assessed on testicular tissues (*ARF*, *CCNA1*, *MGMT*, *MLH1*, *APC* and *RAR-β2*; Muller *et al*, 2000; Koul *et al*, 2002; Honorio *et al*, 2003; Olasz *et al*, 2005; Lind *et al*, 2007), five were not yet tested in this tissue type (*AIM1*, *PGP9.5*, *S100*, *ER-α* and *ER-β*; Fiegl *et al*, 2004; Brait *et al*, 2008; Carvalho *et al*, 2008), and four were recently identified genes by our pharmacological unmasking strategy in different cancer types (*MCAM*, *VGF*, *FKBP4* and *SSBP2*) (Hoque *et al*, 2008).

### DNA extraction

After initial patient de-identification, all original histological slides from the TGCT or normal specimens were reviewed by a pathologist to reconfirm the diagnosis. A representative block was retrieved for DNA extraction. Microdissected NT samples from autopsy material were used for controls. Histological slides from the formalin-fixed, paraffin-embedded tissue were prepared. Slides were microdissected to obtain >50% neoplastic cells. DNA was extracted using standard protocols as previously described (Brait *et al*, 2008). Briefly, DNA was obtained by digestion with 50 *μ*g ml^−1^ proteinase K (Roche, Indianapolis, IN, USA) in the presence of 1% SDS at 48 °C for 2 days, followed by phenol/chloroform extraction and ethanol precipitation, and finally dissolved in 20 μl of LoTE (2.5 mmol l^−1^ EDTA and 10 mmol l^−1^ Tris-HCL) and stored at −20 °C until used.

### Sodium bisulphite treatment

DNA extracted from tumour or normal tissue was subjected to bisulphite treatment with the EpiTect Bisulfite kit (QIAGEN, Valencia, CA, USA), according to the manufacturer's instructions. Treated DNA was stored at −80 °C until used.

### Methylation analysis

Bisulphite-modified DNA was used as a template for fluorescence-based real-time PCR, as previously described (Brait *et al*, 2009). Amplification reactions were carried out in triplicate in a final volume of 20 *μ*l containing 1 *μ*l of bisulfite-modified DNA, 600 nM concentrations of forward and reverse primers, 200 nM probe, 0.6 U of platinum Taq polymerase (Invitrogen, Frederick, MD, USA), 200 μM concentrations each of dATP, dCTP, dGTP and dTTP, and 6.7 mM MgCl_2_. Primers and probes were designed to specifically amplify the promoters of the 15 genes of interest and the promoter of a reference gene, *β-actin*; primer and probe sequences, and annealing temperatures are provided in Supplementary Table 1. Amplifications were carried out using the following profile: 95 °C for 3 min, followed by 50 cycles at 95 °C for 15 s and 60 °C for 1 min. Amplification reactions were carried out in 384-well plates in a 7900HT sequence detector (Applied Biosystems, Foster City, CA, USA) and were analysed by a sequence detector system (SDS 2.3; Applied Biosystems). Each plate included studied DNA samples, positive (*in-vitro* methylated leukocyte DNA) and negative (normal leukocyte DNA or DNA from a known unmethylated cell line) controls and multiple water blanks. Leukocyte DNA from a healthy individual was methylated *in vitro* with excess SssI methyltransferase (New England Biolabs Inc., Beverly, MA, USA) to generate completely methylated DNA, and serial dilutions (90–0.009 ng) of this DNA were used to construct a calibration curve for each plate. All samples were within the assay's range of sensitivity and reproducibility, based on the amplification of the internal reference standard (threshold cycle (CT) value for *β-actin* of 40). The relative level of methylated DNA for each gene in each sample was determined as a ratio of methylation-specific PCR-amplified gene to *β-actin* (reference gene), and then multiplied by 1000 for easier tabulation (average value of triplicates of the gene of interest divided by the average value of triplicates of *β-actin* × 1000).

This measure represents the relative level of methylation in a particular sample and was used for direct comparison of samples. For a tumour sample to be considered methylated at a specific gene, it had to meet two specific criteria. Amplification must have been present in at least two of the three reaction wells in the triplicate run, and the *β-actin*-normalised mean methylation value must have fallen within the range of the serial standard curve dilutions. The lack of DNA contamination was verified by the absence of amplification of a distilled water negative control for each QMSP run.

### Statistical analysis

The primary objective in this study was to describe the methylation patterns of 15 well-established or putative TSGs in NT and two TGCT types, SE and NSE.

Gene methylation was treated as a continuous variable. Univariate and multivariable logistic regression models were constructed sequentially to examine the association of gene methylation with the disease status (tumour *vs* normal). Odds ratios (OR) were estimated with 95% confidence intervals (CI), which quantified the strength of the association and its uncertainty. The final model was selected by using the backward selection method in multivariable logistic regression.

Correlation in methylation between genes was also examined so as to aid in the selection of genes that independently contributed to distinguishing tumour from normal samples (or the two subtypes: SEs and NSEs). Receiver-operating characteristic (ROC) analyses were conducted to evaluate the marker validity to differentiate tumour and normal. Sensitivity, specificity and area under the curve (AUC) were estimated along with 95% CI. Correlated ROC curves were compared using DeLong's method, which is a non-parametric approach (DeLong *et al*, 1988).

On the basis of the correlations among all the 15 genes obtained using a non-parametric approach along with the individual gene performance, the genes shown to be independent of each other by Spearman correlation test, were selected to be included in a logistic regression model. This model was used to assess the probability of tumour. The genes explored further for panel performance were those with *P*<0.10 in the final model. Multi-variable logistic regression was performed with genes of interest, as well as patient characteristics (age, race and site), entered the model simultaneously and were eliminated by backwards selection. An association was considered statistically significant with *P*-value <0.05. All *P*-values reported are two-sided.

The optimal cutoff was determined as the point at which it simultaneously maximised sensitivity and specificity. We used cancer cases and NT samples for generating ROC curves for individual genes, and the cut point of methylation values were established with the values that optimally differentiate the two sample groups. Representative ROC curves are available in Supplementary Figure 1.

## Results

Patients with TGCT were aged between 22 and 62 years (median=33). Age range of controls was 20 to 81 (median=55). Patients with SE were aged between 22 and 62 years (median=34), and for NSE, were between 23 and 38 years (median=27.5). Control subjects were significantly older compared with patients with overall TGCT (*P*=0.05, Wilcoxon non-parametric test). The samples include 46 stage-I TGCT and 11 ⩾stage-II TGCT. Thirty-five TGCT analysed were from right site and 22 from left site. The majority of the samples were obtained from Caucasian. Patient characteristics included in this study are summarised in Table 1.

### Overall methylation frequency in TGCTs

A total of 15 genes (*AIM1*, *ARF*, *CCNA1*, *MGMT*, *hMLH1*, *PGP9.5*, *S100A2*, *APC*, *RAR-β2*, *ER-α*, *ER-β*, *MCAM*, *VGF*, *FKBP4* and *SSBP2*) were analysed for promoter methylation using QMSP in 57 tumour samples, comprising 43 SEs and 14 NSEs, and 23 NT samples. The observed frequencies of each gene using optimal cutoffs that maximised specificity and sensitivity are summarised in Table 2.

*VGF* show a significant cancer-specific methylation (*P*=0.008, by Fisher's exact test). A total of 14 out of 57 TGCT showed methylation of the promoter region of VGF, whereas no methylation was observed in NT. *hMLH1* was significantly methylated in TGCT (39% (22 out of 57)) in comparison with normal (4% (1 out of 23)); *P*=0.002, by Fisher's exact test).

### Methylation in SE and NT

A total of 15 genes (*AIM1*, *ARF*, *CCNA1*, *MGMT*, *hMLH1*, *PGP9.5*, *S100A2*, *APC*, *RAR-β2*, *E-Rα*, *ER-β*, *MCAM*, *VGF*, *FKBP4* and *SSBP2*) was analysed for promoter methylation in 23 NT and 43 SE cases by QMSP. Results for all genes in all SE cases and controls (NTs) are presented in Table 3a. Individual gene sensitivity and specificity was determined on the basis of an empiric cutoff values by maximising sensitivity and specificity, and the AUC values were calculated for each gene. At least one TSG locus was methylated in 37 out of 43 SE cases (86%), and a total of 11 out of 43 (25.6%) tumour samples showed methylation at three or more of the loci. By a non-parametric statistical approach (DeLong's method), we determined the correlation of methylation events among all the 15 genes. By Spearman correlation test, eight genes (*AIM1, CCNA1, MCAM, PGP9.5, hMLH1, ARF, VGF* and *APC*) showed to be independent of each other and were selected to be included in a logistic regression model, which predict the probability of tumour. Patient characteristics (age, race and site) were included in the multi-variable logistic regression model, together with the previously mentioned eight genes. Smoking status and alcohol consumption were not considered in this analysis, as there were too many unknowns reported. Backward selection was used and the significance level of staying in the final model was set at *P*<0.10. Among all eight genes, only *APC* and *MCAM* remained statistically significant and stayed in the final model (*P*=0.057 and *P*=0.035, respectively) after controlling for patient characteristics (age, race and site). We then grouped the independent genes in different combinations (*AIM1, CCNA1, MCAM, PGP9.5, hMLH1, ARF, VGF* and *APC*), considering optimal cutoffs that separated both groups, and established the maximum possible specificity at 78%, and each combination had a slight different sensitivity, with no significant improvement as shown in Table 3b.

### Methylation in NSE and NT

The frequency of promoter methylation, including the cutoff value at each gene included in this panel is listed in Tables 4a. Briefly the methylation frequency were: *AIM1* 21%, *ARF* 0%, *CCNA1* 14%, *MCAM* 71%, *MGMT* 50%, *MLH1* 71%, *PGP9.5* 14%, *S100A2* 57%, *SSBP2* 57%, *APC* 50%, *RAR-β2* 21%, *VGF* 50%, *ER-α* 36%, *ER-β* 64% each and *FKBP4* 36%.

On the basis of the correlations among all the 15 genes obtained using a non-parametric approach along with the individual gene performance, six genes (*APC*, *VGF*, *MGMT*, *hMLH1*, *ER-β* and *FKBP4*) were shown to be independent of each other and each of them shows the strongest predictive potential representing the corresponding cluster, and thus, were selected to be included in a multivariable logistic regression model that modelled the probability of tumour. However, issues arose with such small sample size, that is, for *VGF*, *MGMT* and *FKBP4*, the maximum likelihood estimates are not possible, as there is no variation in methylation values for NT samples (e.g., all normal samples had zero methylation values for each of these three genes). Thus, only *hMLH1*, *APC* and *ER-β* were considered in the multivariable logistic model. It turned out that *hMLH1* remained statistically significant in this final model (*P*=0.044), which indicated its independent effect in predicting tumour. Performance of *hMLH1* combined with *APC* and *ER-β* was explored as well. The significance of *hMLH1* was examined with adjustment for the current available patient characteristics (age, race and site), using the logistic regression model. The independent effect of the genes of interest was evaluated with adjustment for age only. Results indicated that association of *hMLH1* hypermethylation with tumour remained positive (OR=8.29; 95% CI=0.66–103.5), albeit no statistical significance (*P*=0.100). Results should be interpreted with caution with an imprecise estimate for strength of association (i.e., wide CI for the OR estimate). Table 4b shows the predictive performance of the three gene panels (*hMLH1*, *APC* and *ER-β*), considering optimal cutoffs that separated both groups. Supplementary Figure 2 illustrates the predictive powers of hMLH1 alone, as well as combination with *APC* and *ER-β*. There was no statistically significant improvement with the combined genes compared with *hMLH1* alone, based on the non-parametric approach of predictive power of DeLong *et al* (1988).

On the basis of the cutoff value determined for SE cases, six genes (*CCNA1, MGMT, PGP9.5, RAR-β2, VGF* and *FKBP4*) showed 100% specificity (no methylation in NT) in NSE cases. From this six genes, only one gene was methylated in 5 out of 14 (35.7%) cases, two genes were methylated 4 out of 14 (28.5%) cases, one sample showed methylation in three genes, 1 out of 14 (7.1%), and only one sample showed methylation in four genes, 1 out of 14 (7.1%); no samples showed methylation in five genes, and one sample out of 14 (7.1%) had all six genes methylated. At least one of these six genes was observed methylated in 12 out of 14 (86%) of the samples with 100% specificity for NSEs.

### Methylation levels and frequency across sample types

When dividing the groups into normal, SEs and NSEs, one would expect to see a differential methylation of the analysed genes, as methylation is tissue specific and cancer specific. The non-parametric test (DeLong's method) was used to test for differential methylation frequency (in a binary fashion), and five genes met statistical significance (*APC*, *MGMT*, *hMLH1*, *ER-β* and *FKBP4*). This test accounted for the normalised methylation level as a continuous variable. *MGMT*, *VGF*, *CCNA1* and *FKBP4* are interesting, as they could be specific for tumours as all of the normal samples showed no methylation, and in addition, *hMLH1* that had only one positive in normal. There was methylation in normal, SE and NSE in similar frequencies for the following genes: *ARF*, *S100A2*, *SSBP2*, *ER-α* and *ER-β*. Interestingly, *SSBP2* and *ER-α* showed higher levels and frequency of methylation seen in the normal samples than in SE and NSE.

As supported by the previous studies (Peltomaki, 1991; Smiraglia *et al*, 2002; Netto *et al*, 2008), we found differential methylation pattern in SE and NSE. *MGMT, VGF, ER-β* and *FKBP4* are predominately methylated in NSEs when compared with SEs. *APC* and *hMLH1* are shown to be methylated in both subtypes, but *APC* exhibits not only a higher frequency, but also higher levels, and *hMLH1* a significantly higher frequency. When combining *APC, hMLH1, ER-β* and *FKBP4* (considering empiric cutoffs), it is possible to identify 86% of the NSEs, whereas only 7% of the SEs (AUC 0.90 (0.81–1.00)).

**Figure 1:**
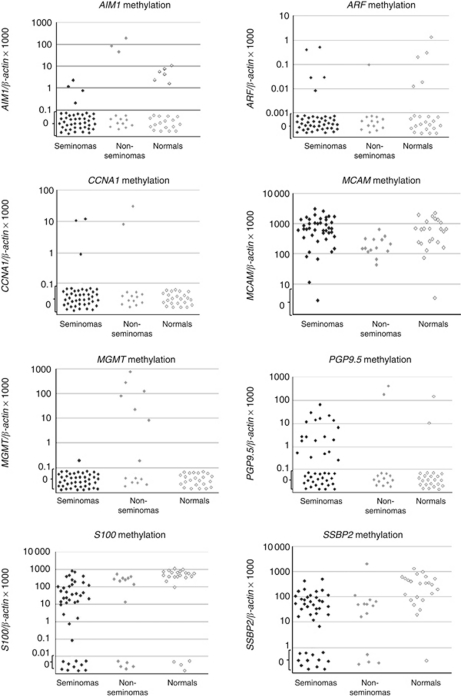

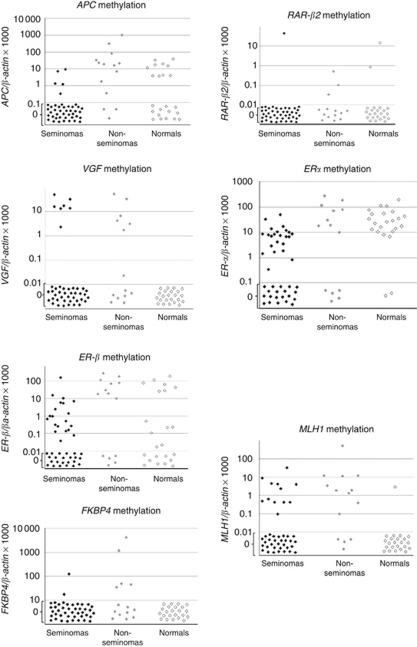
Representative scatter plots of methylation values of tested genes in seminomas, non-seminomas and normal testis samples. Genes: *AIM1*, *ARF*, *CCNA1*, *MGMT*, *hMLH1*, *PGP9.5*, *S100A2*, *APC*, *RAR-β2*, *ER-α*, *ER-β*, *MCAM*, *VGF*, *FKBP4* and *SSBP2.* Calculation of the gene of interest: ratios were based values for both the gene of interest and *β-actin* obtained by quantitative real-time PCR analysis. The obtained ratios were multiplied by 1000 for easier tabulation. Values designated as 0.1 are 0 values, which cannot be plotted correctly on a log scale.

The summary data for the comparison of SEs and NSEs is in Table 5a and b Representative scatter plots of methylation of the 15 genes analysed throughout the testicular tissues are shown in Figure 1.

### Association between DNA methylation changes and clinicopathological factors

There were no apparent correlations between any of the gene tested with any clinicopathological parameters, including age, site, race and disease stages, perhaps due to the limited number of sample size (data not shown). As shown before, we found differential methylation patterns in two different histological types (SEs and NSEs). Methylation values were compared as continuous variables, as well as dichotomised, and no correlation was observed in either analysis.

## Discussion

In this study, we attempted to define a set of methylation markers that would allow for an accurate discrimination among the two most common types of TGCT (SE and NSE); as each type displays dissimilar clinical behaviour and successful pre-operative cytological or histological assessment is restricted. Through gene promoter methylation profiling with QMSP, six genes were found to be differentially methylated in the two tumour types. In particular, higher *PGP9.5* methylation frequency was detected in SEs than NSEs, whereas high methylation frequency of *MGMT, VGF, ER-β* and *FKBP4* were associated with NSE. Remarkably, both SE and NSE were methylated for *APC* and *hMLH1*. Netto *et al* (2008) showed evidence that SEs remain unmethylated, whereas the other histological types arise after *de novo* methylation, suggesting that they may arise in distinct periods of the development process of the germ cells, and that their genome methylation status may determine the degree of differentiation of the cells. Wermann *et al* (2010) also observed the same pattern of all SEs being unmethylated (as well as tumours of similar histology originating in other organs), whereas NSEs were consistently hypermethylated. Koul *et al*, (2002), using conventional MSP, analysed a panel of 21 genes and observed a near absence of methylation in SEs and a higher percentage in NSEs, suggesting a role for different panel of methylation-induced inactivation of TSGs in two common types of TGCT. Similar to our results, they also observed presence of methylation in the *MGMT* promoter in NSEs (21 *vs* 14% observed in this study) and absence in SEs. The other two genes that showed this same pattern were not evaluated by our study (*BRCA1* and *RASSF1A*), and their panel did not include the other genes observed to be methylated in NSEs *vs* SEs (*VGF, ER-β* and *FKBP4*). Subtype-specific patterns of global methylation were also previously reported in TGCT (Smiraglia *et al*, 2002). Peltomaki (1991) observed hypomethylation in SEs, whereas NSEs were largely hypermethylated. The same observation was made in studies of the X chromosome (Looijenga *et al*, 1997). In breast cancer, Lehmann *et al* (2002) observed that the presence of promoter methylation in *DAPK* was frequent in invasive lobular cancer (53%) and not frequent in another histological subtype (9% invasive ductal carcinoma). Other reports that include different epigenetic alterations in histological subtypes of the same cancer type include: presence of methylator phenotype in low-grade gliomas when compared with *de novo* glioblastomas (Laffaire *et al*, 2011), high frequency of *SFN* methylation in small cell lung cancer, whereas a rare frequency in non-small cell lung cancer (Osada *et al*, 2002). Consistent with epigenetic heterogeneity, there is a wide search for genetic alterations that would be able to distinguish SEs and NSEs. Coffey *et al* (2008) reported that Kit mutations are predominant in SEs and very rarely observed in NSEs. Major accentuated genetic differences are yet to be discovered.

Previous studies did indicate a role for hypermethylation of TSG promoters in the pathogenesis of SE. In our data, when comparing SEs versus NT samples, the genes that showed higher frequency of methylation in SE were *PGP9.5*, *hMLH1* and *ER-β*, when compared with the observed frequency in normal samples. In combination, the strongest pair is *APC* and *MCAM*, showing 78.3% frequency in tumours and 23.3% in normals. With the same cutoff, adding *hMLH1*, the frequency in tumours was 79.1%, and adding both *AIM1* and *PGP9.5*, or only one of them, it increases to 81.4%, with the same percentage in normals (23.3%). This panel containing four genes showed reasonable percentages to distinguish normal and tumour tissue.

*MCAM* had not been previously evaluated in TGCT. In prostate cancer, other showed 85% methylation in tumours *vs* 0% in normal prostate (Hoque *et al*, 2008). To our knowledge, *PGP9.5* and *AIM1* had not been analysed in this tumour type. *APC* was previously observed with 10% of methylation in tumours and 0% in NT, but both positive tumours were NSE (Koul *et al*, 2002), and no *APC* methylation has been reported in Ses; it is important to emphasise that this study was performed using conventional MSP, a less sensitive technique than QMSP used in the present study (Hoque *et al*, 2004), which may explain the different percentages observed. In this same study, *hMLH1* showed only 4% methylation in tumours and 0% in normals, occurring in both SEs and NSEs.

In respect to the comparison between NSEs and normals, there are two genes (*MGMT* and *VGF*) that stand out alone, both with a 0% frequency in normals and 50% frequency in NSEs. *VGF* is a gene recently reported to be methylated in cancer by our group (Hoque *et al*, 2008) and had never been analysed in TGCT. *MGMT* had been reported to be frequently methylated in TGCT (Smith-Sorensen *et al*, 2002), showing 69% in NSEs. In 2004, Koul *et al* (2004) showed methylation in *MGMT* in 20% of NSEs analysed by conventional MSP, a less sensitive technique as mentioned earlier. Using empiric cutoffs, at least one of the three genes (*hMLH1, APC* and *ER-β*) was methylated in 13 out of 14 (93%) of NSE cases and 10 out of 23 (43%) of the NT samples. Previously reported methylation of *APC* and *hMLH1* were 10% to less than 5%, respectively (Koul *et al*, 2002). There are no reported studies exploring methylation status of *ER-β* in TGCT. The downregulation of ER*β* was observed in SEs by Esposito *et al* (2010); they detected decreased expression of ER*β* protein in SEs when compared with NT, but they did not evaluate promoter methylation. This gene encodes a member of the family of oestrogen receptors and superfamily of nuclear receptor transcription factors. The gene product contains an N-terminal DNA-binding domain and C-terminal ligand-binding domain, and is localised to the nucleus, cytoplasm and mitochondria. It is able to interact with specific DNA sequences to activate transcription. Some isoforms dominantly inhibit the activity of other oestrogen receptor family members.

Epigenetic inactivation of *hMLH1* is found in a wide range of cancers. *hMLH1* is a DNA mismatch repair (MMR) gene and is an essential component of the DNA MMR pathway, and is frequently mutated in hereditary non-polyposis colon cancer also known as Lynch syndrome. Activation of the MMR pathway may trigger DNA damage signalling, a process which induces cell cycle arrest and can lead to cell death in case of major DNA damages (for review, please see Jiricny (2006)). *hMLH1* is the most prominent target of epigenetic silencing in the MMR pathway in sporadic tumours, comprising ovarian, head and neck, breast and colorectal cancer (Herman *et al*, 1998). However, hypermethylation of *hMLH1* is often associated with other hypermethylated genes, which complicates mechanistic interpretation of associations with response to therapy in patients (Shen *et al*, 2007). Mechanistic investigations using *in vitro* system have shown that treatment of *hMLH1*-methylated colon cancer cell lines with the demethylating agent 5aza-2deoxycytidine (5-aza-dC) restores hMLH1 expression and subsequently renders the cells MMR proficient (Herman *et al*, 1998).

Promoter methylation of *hMLH1* has been associated with chemoresistance to cisplatin-based therapies in ovarian cancer more than a decade ago (Strathdee *et al*, 1999). In our study, both SEs and NSEs displayed methylation in this locus, 27% and 71%, respectively. It is well established that NSE and SE (all TGCT) are widely responsive to cisplatin-based therapy (Bosl and Motzer, 1997), so one wouldn’t expect the high presence of *hMLH1* methylation in this tumour type. Olasz *et al* (2005) hypothesised that this epigenetic alteration could be linked to the chemoresistance in a small group of testicular tumours, but could not observe any association. On the other hand, Honecker *et al.* (2009) investigated a larger cohort and observed that lack or low expression of hMLH1 (on the protein level) was significantly associated with cisplatin-resistant TGCTs, and the absence of expression was also correlated with presence of *hMLH1* promoter methylation. Probably the mechanisms of resistance/sensitivity to this chemothepeutic agent are different among the ovarian and TGCT. It is widely accepted that hMLH1 dysfunction induces microsatellite instability (MSI) in various cancers, and loss of hMLH1 expression was related to its promoter methylation. However, no correlation was observed between *hMLH1* methylation and MSI in TGCTs (Olasz *et al*, 2005). In our study, *hMLH1* methylation was detected in 22 out of 57 (39%) of TGCTs. However, we did not perform expression or MSI analysis in these samples; therefore, we are not able to correlate *hMLH1* methylation with expression and MSI. Esteller *et al* (2000) reported that promoter methylation of *MGMT* results in enhanced sensitivity to alkylating agents in gliomas. In the present study, *MGMT* was highly methylated in NSEs, which could indicate that the epigenetic silencing of this gene may be also linked to the cisplatin-based chemotherapy response, but this data needs to be further explored in a well-defined cohort consisting of cisplatin responsive group and non-responsive group.

Silencing TSG by DNA promoter methylation is an important feature of cancer. It is known that cancer types may vary in their epigenetic profiles in a tissue-specific pattern (Esteller *et al*, 2001). We used a candidate gene approach to test 15 promoter regions in a testicular cohort; all the markers evaluated here have shown differential methylation in malignant *vs* benign tissues in several cancer types (Hoque *et al*, 2008). Our data shows different patterns of methylation not only between tumours and normal, but also between the two histological subtypes of germ cell tumours. These distinct promoter methylation profiles of the two main subtypes of germ cell testicular cancer, SEs and NSEs, may shed a light on how they develop and differentiate from the same cell of origin. In conclusion, our study constitutes a comprehensive profile of hypermethylated genes in testicular tissues. Several of genes tested in this study were not evaluated for promoter methylation in this cancer types before, which may partially be due to rarity of the disease and limited number of samples. The observation of the higher proportion of promoter methylation of putative and established TSGs in NSEs when compared with SEs may enrich our knowledge of this tumour type. Further study using larger cohorts is needed to evaluate the potential use of these methylation markers in the detection, prognosis and therapeutic outcome of this rare tumour.

## Figures and Tables

**Table 1:** Demographic and clinical characteristics of testicular cancer patients (*N*=57, seminomas=43, non-seminomas=14) and normals (*N*=24)

**Characteristic**	**Number of patients (%)**		
**Hystologic diagnosis**	**Seminoma**	**Non-seminoma**	**Normal**
Total samples	43 (100)	14 (100)	23 (100)
			
*Site*			
Right	25 (58.1)	10 (71.4)	5 (21.7)
Left	18 (41.9)	4 (28.6)	3 (13.1)
Unknown	0 (0)	0 (0)	15 (65.2)
			
*Age (years)*			
Median age (range)	34 (22–62)	27.5 (23–38)	55 (20–81)
			
*Race*			
Caucasian	35 (81.4)	10 (71.4)	18 (78.3)
African American	4 (9.3)	2 (14.2)	5 (21.7)
Others	4 (9.3)	3 (21.4)	0 (0)
			
*Stage*			
I	40 (93.0)	6 (42.9)	
II	3 (7.0)	5 (35.7)	N/A^a^
III	0 (0)	3 (21.4)	

a N/A=Not applicable.

**Table 2:** Promoter methylation frequency in TGCT and in normal testicular samples

**Genes**	**Cutoff** a	**Number of tumors with methylation/total number of tumors (%, 95% CI)**	**Number of tumors with methylation/total number of tumors (%, 95% CI)**
*AIM1*	44.817	3/57 (5, 1.1–14.6)	1/23 (4.3, 0.1–22.0)
*ARF*	0.3	2/57 (3.5, 0.4–12.1)	2/23 (8.7, 1.1–28.0)
*CCNA1*	8.173	5/57 (8.8, 2.9–19.3)	0/23 (0, 0–14.8)
*MCAM*	1073	23/57 (40.4, 27.6–54.2)	9/23 (39.1, 19.7–61.5)
*MGMT*	0.193	8/57 (14, 6.3–25.8)	0/23 (0, 0–14.8)
*MLH1*	0.096	22/57 (38.6, 26.0–52.4)	1/23 (4, 0.1–22.0)
*PGP9*	177.09	2/57 (3.5, 0.4–12.1)	0/23 (0, 0–14.8)
*S100*	858.15	23/57 (40.4, 27.6–54.2)	19/23 (83, 61.2–95.1)
*SSBP2*	623.99	8/57 (14, 6.3–25.8)	16/23 (69.5, 47.1–86.8)
*APC*	19.077	7/57 (12.3, 5.1–23.7)	19/23 (83, 61.2–95.1)
*RAR-β2*	14.273	1/57 (1.8, 0–9.4)	0/23 (0, 0–14.8)
*VGF*	0.023	14/57 (24.6, 14.1–37.8)	0/23 (0, 0–14.8)
*ER-α*	72.072	6/57 (10.5, 4.0–21.5)	10/23 (43.5, 23.2–65.5)
*ER-β*	10.145	13/57 (22.8, 12.7–35.8)	7/23 (30, 13.2–52.9)
*FKBP4*	35.001	7/57 (12, 5.1–23.7)	0/23 (0, 0–14.8)

Abbreviations: CI=confidence intervals; TGCT=testicular germ cell tumors.

a The optimal cutoff was determined as the point at which it simultaneously maximized sensitivity and specificity.

**Table 3a:** Seminomas and normal testicular samples: (a) promoter methylation frequency in seminomas and normal testicular samples

		**Seminoma**	**Normal**
**Genes**	**Cutoff**	**Number of tumors with methylation/total number of tumors (%, 95% CI)**	**Number of tumors with methylation/total number of tumors (%, 95% CI)**
*AIM1*	44.817	0/43 (0, 0–8.2)	1/23 (4.3, 0.1–22.0)
*ARF*	0.3	5/43 (11.6, 3.9–25.1)	2/23 (8.7, 1.1–28.0)
*CCNA1*	8.173	2/43 (4.6, 0.6–15.8)	0/23 (0, 0–14.8)
*MCAM*	1073	14/43 (32.6, 19.1–48.5)	9/23 (39.1, 19.7–61.5)
*MGMT*	0.193	1/43 (2.3, 0.1––12.3)	0/23 (0, 0–14.8)
*MLH1*	0.096	12/43 (27.9, 15.3–43.7)	1/23 (4, 0.1–22.0)
*PGP9.5*	177.09	4/43 (9.3, 2.6–22.1)	0/23 (0, 0–14.8)
*S100*	858.15	0/43 (0, 0–8.2)	19/23 (83, 61.2–95.1)
*SSBP2*	623.99	0/43 (0, 0–8.2)	16/23 (69.5, 47.1–86.8)
*APC*	19.077	5/43 (11.6, 3.9–25.1)	19/23 (83, 61.2–95.1)
*RAR-β2*	14.273	1/43 (2.3, 0.1–12.3)	0/23 (0, 0–14.8)
*VGF*	0.023	7/43 (16.3, 6.8–30.7)	0/23 (0, 0–14.8)
*ER-α*	72.072	8/43 (18.6, 8.4–33.4)	10/23 (43.5, 23.2–65.5)
*ER-β*	10.145	12/43 (27.9, 15.3–43.7)	7/23 (30, 13.2–52.9)
*FKBP4*	35.001	1/43 (2.3, 0.1–12.3)	0/23 (0, 0–14.8)

**Table 3b:** Seminomas and normal testicular samples: (b) analysis of gene panels, based on logistic regression model, considering optimal cutoff for differenciating both groups

**Panel**	**Cutoff based on predictive probability of tumor** a	**Sensitivity, % (95% CI)**	**Specificity, % (95% CI)**	**AUC (95% CI)**
*MCAM, APC*	0.643971	76.7 (61.4–88.2)	78.3 (56.3–92.5)	0.81 (0.70–0.92)
*MCAM, APC, MLH1*	0.620704	79.1 (64.0–90.0)	78.3 (56.3–92.5)	0.84 (0.74–0.94)
*MCAM, APC, MLH1, AIM1*	0.601593	81.4 (66.6–91.6)	78.3 (56.3–92.5)	0.85 (0.75–0.95)
*MCAM, APC, MLH1, AIM1, PGP9.5*	0.590917	81.4 (66.6–91.6)	78.3 (56.3–92.5)	0.86 (0.76–0.95)

Abbreviations: AUC=area under the curve; CI=confidence interval.

a Predictive probabilities were obtained from the logistic regression models.

**Table 4a:** Non-seminomas and normal testicular samples: (a) promoter methylation frequency in non-seminomas and normal testicular samples

		**Non-seminoma**	**Normal**
**Genes**	**Cutoff**	**Number of tumors with methylation/total number of tumors (%, 95% CI)**	**Number of tumors with methylation/total number of tumors (%, 95% CI)**
*AIM1*	44.817	3/14 (21.4, 4.7–50.8)	1/23 (4.3, 0.1–22.0)
*ARF*	0.3	0/14 (0, 0- 6.9)	2/23 (8.7, 1.1–28.0)
*CCNA1*	8.173	2/14 (14.3, 1.8–42.8)	0/23 (0, 0–14.8)
*MCAM*	1073	10/14 (71.4, 41.9–91.6)	9/23 (39.1, 19.7–61.5)
*MGMT*	0.193	7/14 (50, 23.0–77.0)	0/23 (0, 0–14.8)
*MLH1*	0.096	10/14 (71.4, 41.9–91.6)	1/23 (4, 0.1–22.0)
*PGP9*	177.09	2/14 (14.3, 1.8–42.8)	0/23 (0, 0–14.8)
*S100*	858.15	8/14 (57.1, 28.9–82.3)	19/23 (83, 61.2–95.1)
*SSBP2*	623.99	8/14 (57.1, 28.9–82.3)	16/23 (69.5, 47.1–86.8)
*APC*	19.077	7/14 (50, 23.0–77.0)	19/23 (83, 61.2–95.1)
*RAR-β2*	14.273	3/14 (21.4, 4.7–50.8)	0/23 (0, 0–14.8)
*VGF*	0.023	7/14 (50, 23.0–77.0)	0/23 (0, 0–14.8)
*ER-α*	72.072	5/14 (35.7, 12.8–64.9)	10/23 (43.5, 23.2–65.5)
*ER-β*	10.145	9/14 (64.3, 35.1–87.2)	7/23 (30, 13.2–52.9)
*FKBP4*	35.001	5/14 (35.7, 12.8–64.9)	0/23 (0, 0–14.8)

Abbreviation: CI=confidence interval.

**Table 4b:** Non-seminomas and normal testicular samples: (b) analysis of gene panels, based on logistic regression model, considering optimal cutoff for differentiating both groups

**Combined genes**	**Cutoff based on predictive probability of tumor** a	**Sensitivity (%)**	**Specificity (%)**	**AUC (95% CI)**
*MLH1, APC, and ER-β*	0.225668	85.7 (57.2–98.2)	82.6 (61.2–95.1)	0.89 (0.75 – 1.00)

Abbreviations: AUC=area under the curve; CI=confidence interval.

a Predictive probabilities were obtained from the logistic regression models.

**Table 5a:** Seminomas and non-seminoma testicular samples: (a) promoter methylation frequency in seminomas and non-seminoma testicular samples

		**Non-seminoma**	**Seminoma**
**Genes**	**Cutoff**	**Number of tumors with methylation/total number of tumors (%, 95% CI)**	**Number of tumors with methylation/total number of tumors (%, 95% CI)**
*AIM1*	44.817	3/14 (21.4, 4.7–50.8)	0/43 (0, 0–8.2)
*ARF*	0.3	0/14 (0, 0–6.9)	5/43 (11.6, 3.9–25.1)
*CCNA1*	8.173	2/14 (14.3, 1.8–42.8)	2/43 (4.6, 0.6–15.8)
*MCAM*	1073	10/14 (71.4, 41.9–91.6)	14/43 (32.6, 19.1–48.5)
*MGMT*	0.193	7/14 (50, 23.0–77.0)	1/43 (2.3, 0.1–12.3)
*MLH1*	0.096	10/14 (71.4, 41.9–91.6)	12/43 (27.9, 15.3–43.7)
*PGP9*	177.09	2/14 (14.3, 1.8–42.8)	4/43 (9.3, 2.6–22.1)
*S100*	858.15	8/14 (57.1, 28.9–82.3)	0/43 (0, 0–8.2)
*SSBP2*	623.99	8/14 (57.1, 28.9–82.3)	0/43 (0, 0–8.2)
*APC*	19.077	7/14 (50, 23.0–77.0)	5/43 (11.6, 3.9–25.1)
*RAR-β2*	14.273	3/14 (21.4, 4.7–50.8)	1/43 (2.3, 0.1–12.3)
*VGF*	0.023	7/14 (50, 23.0–77.0)	7/43 (16.3, 6.8–30.7)
*ER-α*	72.072	5/14 (35.7, 12.8–64.9)	8/43 (18.6, 8.4–33.4)
*ER-β*	10.145	9/14 (64.3, 35.1–87.2)	12/43 (27.9, 15.3–43.7)
*FKBP4*	35.001	5/14 (35.7, 12.8–64.9)	1/43 (2.3, 0.1–12.3)

Abbreviation: CI=confidence interval.

**Table 5b:** Seminomas and non-seminoma testicular samples: (b) analysis of gene panels, based on logistic regression model, considering optimal cutoff for differenciating both groups

**Combined genes**	**Cutoff based on predictive probability of tumor** a	**Sensitivity (%)**	**Specificity (%)**	**AUC (95% CI)**
*APC*	0.3644	78.6 (49.2–95.3)	90.7 (77.9–97.4)	0.87 (0.74–0.99)
*APC, MLH1, ER-β and FKBP4*	0.124726	85.7 (57.2–98.2)	93.0 (80.9–98.5)	0.90 (0.81–1.00)

Abbreviations: AUC=area under the curve; CI=confidence interval.

a Predictive probabilities were obtained from the logistic regression models.
