# Supplementary material for: DNA methylation profiles delineate epigenetic heterogeneity in seminoma and non-seminoma
Source: Br J Cancer. 2011 Nov 8;106(2):414–23. doi: 10.1038/bjc.2011.468 (PMC3261686; doi:10.1038/bjc.2011.468)
Supplement: Supplementary Figures 1 and 2 [file bjc2011468x1.ppt]

## Slide 1
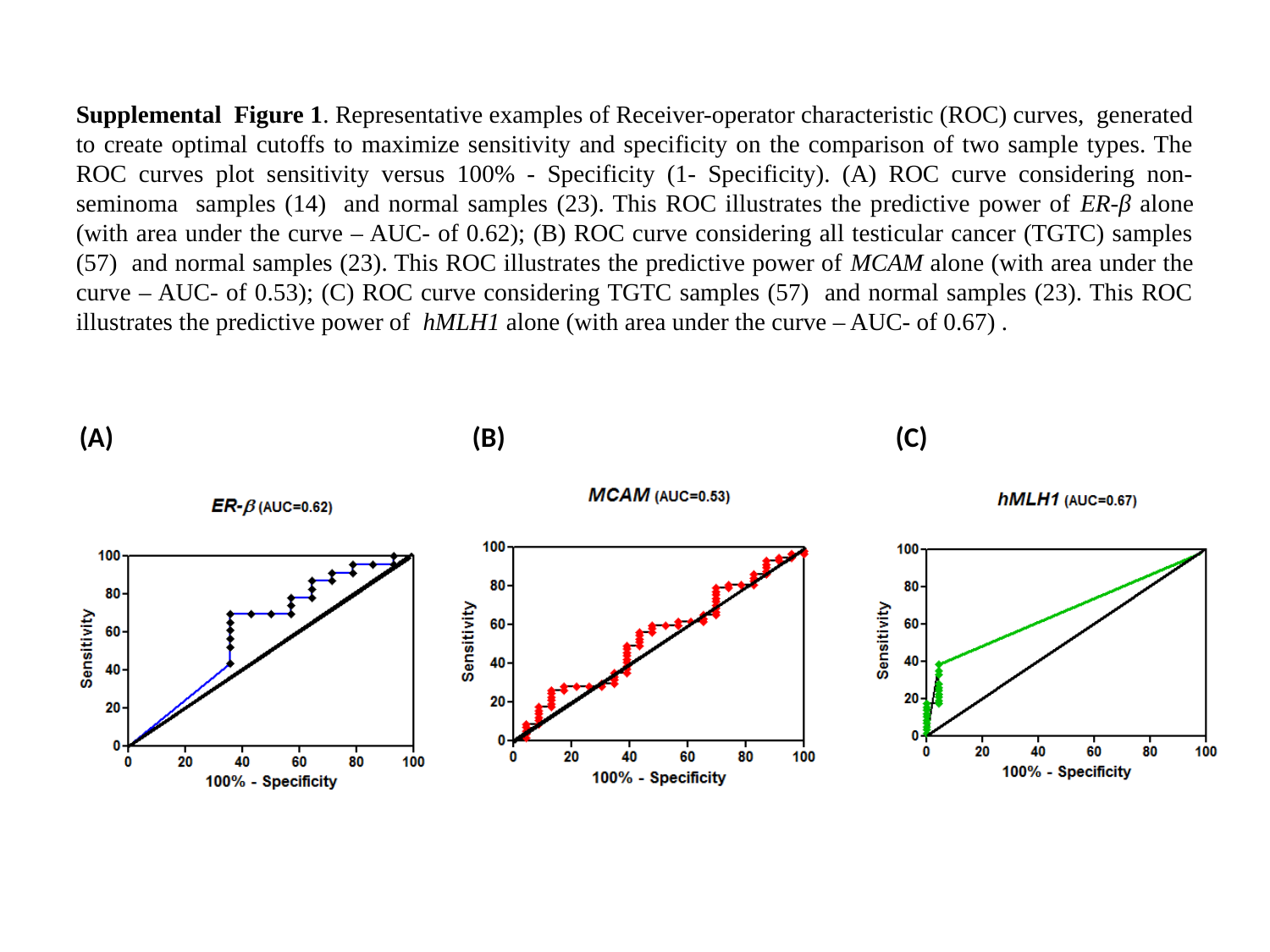

# Supplemental Figure 1. Representative examples of Receiver-operator characteristic (ROC) curves, generated to create optimal cutoffs to maximize sensitivity and specificity on the comparison of two sample types. The ROC curves plot sensitivity versus 100% - Specificity (1- Specificity). (A) ROC curve considering non-seminoma samples (14) and normal samples (23). This ROC illustrates the predictive power of ER-β alone (with area under the curve – AUC- of 0.62); (B) ROC curve considering all testicular cancer (TGTC) samples (57) and normal samples (23). This ROC illustrates the predictive power of MCAM alone (with area under the curve – AUC- of 0.53); (C) ROC curve considering TGTC samples (57) and normal samples (23). This ROC illustrates the predictive power of hMLH1 alone (with area under the curve – AUC- of 0.67) .
(A)
(B)
(C)

## Slide 2
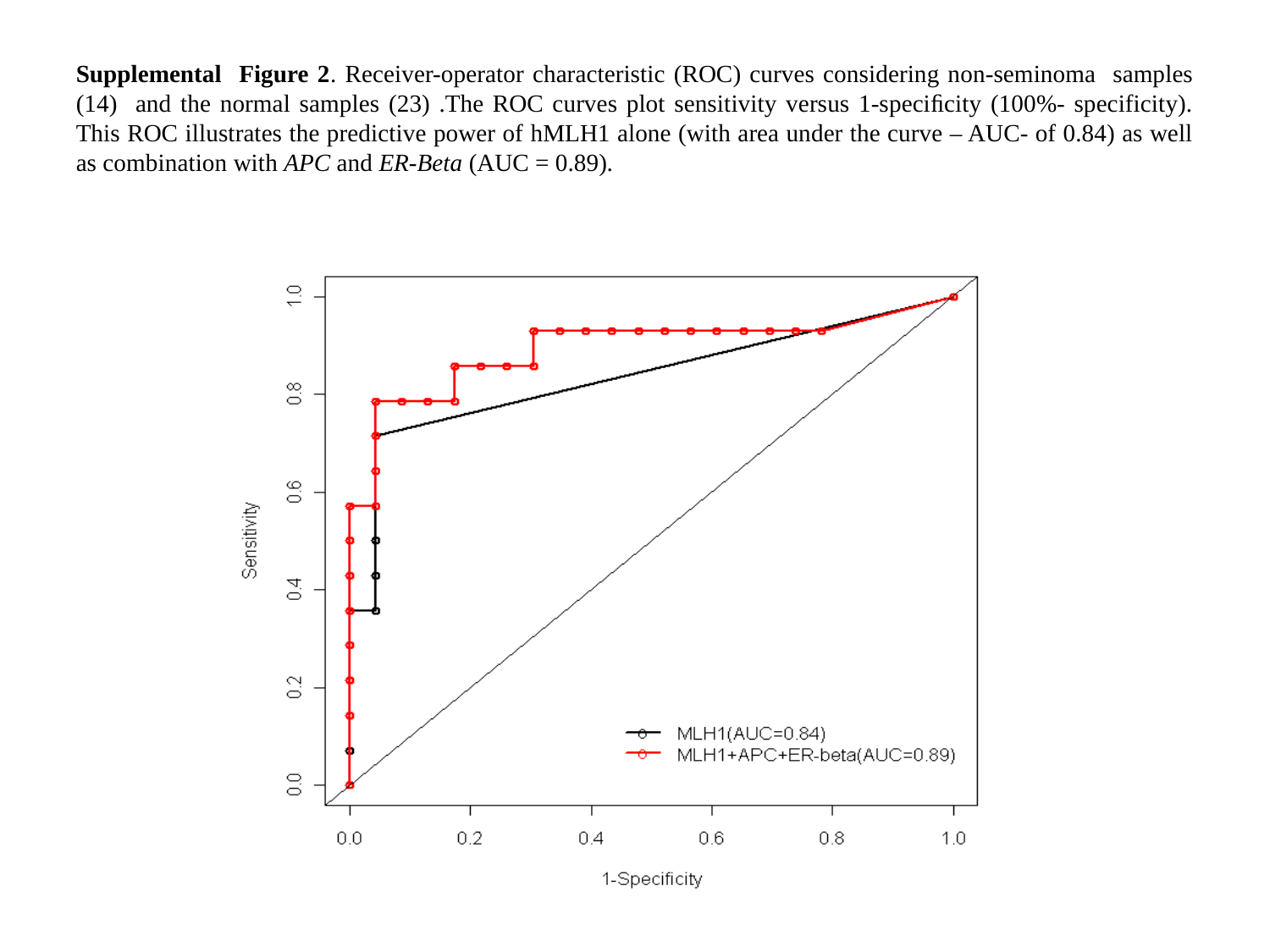

# Supplemental Figure 2. Receiver-operator characteristic (ROC) curves considering non-seminoma samples (14) and the normal samples (23) .The ROC curves plot sensitivity versus 1-speciﬁcity (100%- specificity). This ROC illustrates the predictive power of hMLH1 alone (with area under the curve – AUC- of 0.84) as well as combination with APC and ER-Beta (AUC = 0.89).
